# Serious Complications and Treatment Strategies Associated with Odontogenic Infections

**DOI:** 10.5152/eurasianjmed.2023.23378

**Published:** 2023-12-01

**Authors:** Ebru Acar Evsen, Merve Candan, Merve Pelin Dur

**Affiliations:** 1 Department of Pedodontic Dentistry, Eskişehir Osmangazi University Faculty of Dentistry, Eskişehir, Turkey; 2 Department of Restorative Dentistry, Sakarya University Faculty of Dentistry, Sakarya, Turkey

**Keywords:** Cavernous sinus thrombosis, cerebral abscess, Descending necrotizing mediastinitis, necrotizing fasciitis, odontogenic infections, orbital cellulitis

## Abstract

Dental caries is a prevalent chronic disease on a global scale, affecting individuals at some point in their lives. Odontogenic infections (OIs) arise from dental origins and can be caused by several pathologies, such as caries, trauma, pericoronitis, gingivitis, and iatrogenic factors. Dental caries is a prevalent chronic disease on a global scale, affecting individuals at some point in their lives. The objective of this review is to provide clinicians with information regarding uncommon yet severe complications that can arise from OIs. These complications include necrotizing fasciitis, cerebral abscess, orbital cellulitis, descending necrotizing mediastinitis, sepsis, and cavernous sinus thrombosis. The review encompasses the origin of complications, the progression of infection, as well as their diagnosis and treatment. Early diagnosis and treatment are crucial for determining the clinical course of odontogenic infections. Treatment and diagnosis of OIs in a timely manner prevent complications. Dental professionals play a critical role in the prevention and assessment of OIs, particularly those that are progressive and difficult to treat and identify.

**Main Points**

- These are rare but serious complications that may arise from OIs. These complications include necrotizing fasciitis, cerebral abscess, orbital cellulitis, descending necrotizing mediastinitis, sepsis, cavernous sinus thrombosis, and Lemierre’s syndrome.
- Early diagnosis and treatment are important in preventing OI. Consultation, advanced imaging methods, and bacterial culture studies may be required for accurate diagnosis.
- Dental professionals play a critical role in the prevention and assessment of OIs.

## Introduction

Orofacial infections are prevalent pathologies in dental practice. These infections are classified as odontogenic and non-odontogenic infections. Odontogenic infections (OIs) arise from dental origins and can be caused by several pathologies, such as trauma, caries, pericoronitis, gingivitis, and iatrogenic factors.^1^ Odontogenic infections can be disseminated or localized. A simple incision, drainage, and examination of the infection’s dental origin are all viable methods for managing localized infections. Severe OIs are identified when these infections invade the fascial spaces of the head and neck; in such cases, an operating room incision and drainage are required.^2^

Infections of the deep neck cavity impact various cervical cavities, beginning with the upper respiratory or digestive system.^3^ The prevalence of deep neck cavity infections (DNCIs), which especially affect the perimandibular region, is high, and its etiological and therapeutic aspects are still debated today. However, DNCIs, life-threatening infections that spread rapidly, have been reported infrequently in the scientific literature. Dental infections are among the most prevalent causes of DNCIs. Additionally, pharyngeal and tonsil-related infections and upper respiratory tract infections are among the causes of DNCIs.^4^ A dental origin for infection is frequently an infection of the second or third mandibular molar. The unpredictable progression of the infection frequently depends on the tooth that caused it and the morphology of the individual’s head and neck.

The aim of the present review is to inform clinicians about the rare serious complications of OIs and their treatment strategies.

## Clinical and Research Consequences: Complications Associated with Severe Odontogenic Infections

### Descending Necrotizing Mediastinitis

Descending necrotizing mediastinitis (DNM) is a potentially fatal and aggressive form of DNCI characterized by the necrosis of soft tissues, including muscle, fascia, and fat. It is caused by an oropharyngeal or cervical infection. Deep neck cavity infections progress toward the mediastinal spaces under the influence of gravity and negative intrathoracic pressures caused by breathing.^5^ Serious and potentially lethal, this infection is notable for a substantial mortality rate attributable to complications such as sepsis and infection. Sepsis develops rapidly in the presence of a mediastinal abscess, which frequently invades the mediastinal connective tissues in an aggressive manner.^6^ Similar to DNCIs, infections of the mandibular second and third molars account for over half of DNM cases.^7^ Intensive care units are necessary for the treatment of the preponderance of patients.^8^

According to pathophysiology, polymicrobial infections are to blame for about 58% of cases of DNM. In the remaining cases, only gram-positive bacteria can pose a threat, such as anaerobes or streptococci. *Klebsiella* and other gram-negative Enterobacteriaceae may also serve as causative agents in diabetic patients.^9^

Diagnostic criteria for DNM are defined as follows:^10^

1. The clinical signs and symptoms of a clinically severe oropharyngeal infection;
2. The radiographic findings of mediastinitis by computed tomography;
3. The presence of mediastinal infection detected during an operation or autopsy;
4. The association between oropharyngeal infection and DNM.

Fever, dysphagia, and odynophagia; swelling or stiffness in the neck and upper chest; chest pain; neck tension; dyspnea; respiratory failure; and hypoxia are typical symptoms of mediastinal involvement due to infection. However, symptoms may not always be obvious and may be observed more frequently in the later stages of the infection. Due to the difficulty of early clinical diagnosis, DNCIs extending down to the mediastinum have high mortality rates.^11^

As a result, radiological examination, a fundamental noninvasive diagnostic technique, is applied frequently.^7,11^ Computed tomography (CT) examinations possess considerable diagnostic utility owing to their high sensitivity in identifying the initial signs of mediastinitis.^12^

Patients with mediastinitis require ongoing assessment and management within the intensive care unit. In general, acute mediastinitis is managed through a combination of surgical debridement and prompt initiation of antibiotics, according to the approach of professionals.^13^ Antibiotic selection should be broad spectrum and then culture specific. Second- and third-generation cephalosporins and metronidazole are frequent choices for initial empiric therapy.^11^ Various surgical techniques exist to treat mediastinitis, ranging from minimally to maximally invasive. The selection of an appropriate technique is contingent upon the degree and severity of the condition. Frequent reassessment of the patient’s clinical picture, close laboratory monitoring, and repeat CT should be performed after surgical intervention to determine whether additional surgery is necessary.^13^

### Airway obstruction

Airway obstruction (AO) is a feared complication of severe OIs. The AO and deviation may become substantial when these infections spread to the deep fascial spaces. Ludwig’s angina, which particularly affects the submental space as well as bilateral submandibular and sublingual spaces, is a serious infection that causes AO. It has been reported that OI is more likely to affect the airway than DNCI caused by nonodontogenic causes.^14^ It is generally a polymicrobial infection, with *Streptococcus*, *Bacteroides*, and *Staphylococcus* being the causative microorganisms.^15^

The infection manifests clinically as hoarseness, pain, saliva, dysphonia, and edema of the muscle tissue in the neck. As a result of the aforementioned fascial spaces being involved, the tongue is frequently forced backward and upward, which causes AO.^2^ A number of these symptoms impede the ability to maintain an open airway. In addition, deterioration of anatomy and immobility of tissues are among the factors that make airway management difficult with appropriate intubation.^2^ When intubation is anticipated to be challenging, a video laryngoscope or fiberscope equipped with a camera capable of generating real-time images is utilized. Failure to select the appropriate intubation method will necessitate a repetition of the intubation process. A secondary edema accompanied by pharyngeal or laryngeal injury may result from improper intubation-induced hemorrhage.^16^ Before intubation, it is crucial to assess the circumstances surrounding the airway in a careful and detailed manner.

A study reported that methods such as video-assisted laryngoscopy or flexible bronchoscopy are helpful in airway obstructions resulting from odontogenic infection.^2^ In a study conducted in patients with severe odontogenic infection, video-assisted laryngoscopes were reported to be 100% successful, while traditional tracheal intubation showed a 34% failure.^17^ In a case report describing airway obstruction due to an infection spreading from the submandibular space, it was observed that the patient’s symptoms decreased with the rapid start of antibiotics, but no corticosteroids were used.^18^ No definitive conclusion has been reached in the literature regarding the use of corticosteroids for airway obstruction. While there are studies stating that the use of corticosteroids makes the penetration of antibiotics easier by reducing edema in the area, there are also sources stating that the use of this drug group should be limited.^19,20^

Trismus is a late finding in DNCI cases affecting the airway, such as Ludwig’s angina. While CT is indicated in patients who have not yet developed trismus and can tolerate lying supine, ultrasonography can be used as an appropriate imaging method in patients who cannot tolerate lying down. If definitive airway management is necessary due to the risk of AO, emergency anesthesia and consultation with an otolaryngologist may be beneficial. The first step for airway intervention in the emergency department is endoscopy with flexible intubation along with surgical airway preparation. Broad-spectrum antibiotics and surgical control of the source of infection are key to the treatment of the infection. These patients should then be hospitalized in the intensive care unit for close observation of the airway.^19^

### Orbital Cellulitis/Abscess

Cellulitis, or abscess of the orbit, is frequently caused by bacterial rhinosinusitis. A minority of orbital abscess cases are caused by facial, middle ear, tonsil, or dental infections.^2^ Severe OIs involving the orbit are a rare but serious complication. It can be induced by osteitis, apical periodontal disease, or an infected alveolar socket. OIs typically infiltrate the orbit via the associated vasculature, pterygoid venous plexus, maxillary sinus, and infratemporal or pterygopalatine fossa.^21^

*Streptococcus anginosus*, an increasingly frequently isolated member of the *Streptococcus milleri* group, a commensal organism in the oropharynx, can cause odontogenic abscesses that produce severe gas in adjacent or distant areas. Gaseous orbital cellulitis, which occurs when *S. anginosus* infections originating from teeth spread to the orbit, has been reported by the authors.^21^ In the case reported by Rothschild et al., it has been shown with the help of CT that it reaches the pterygopalatine fossa from the temporalis muscle and then to the orbit through the inferior orbital fissure.^21^ Orbital cellulitis caused by odontogenic sources may result in severe complications, including blindness, necrotizing fasciitis, optic neuropathy, brain abscess, or vascular occlusion.^22,23^ Vision loss may occur in relevant patients; subsequent to the advent of antibiotic therapies, this percentage has fallen below 10%.^24^

Treatment for orbital cellulitis depends on the severity of the infection and its extent of spread. Even though conservative medical treatment methods are preferred, there is a 20% complication rate.^25^ Orbital cellulitis treatment should be done with antibiotics and other supportive treatments. Broad-spectrum antibiotics that will affect *S. aureus, Streptococcus pneumoniae*, other Streptococci, and gram-negative bacilli should be preferred. When a possible intracranial spread is suspected, the antibiotics chosen should also be effective against anaerobic bacteria.^26^

In advanced cases where the response to medical treatment is not sufficient, surgical intervention is recommended. It is critical to eliminate the dental focus, which serves as the source of the infection, in order to effectively treat OIs.^27^ Previously, peri-orbital incisions were used for draining the abscess; however, in the current day, sinus/nasal endoscopic/computer-assisted procedures are utilized instead of facial incisions.^28^

### Septic Cavernous Sinus Thrombosis

Cavernous sinuses are dural sinuses located within the area defined as the lateral sellar compartment and are responsible for the transfer of venous blood from the cranial cavity.^29^ Trochlear, oculomotor, maxillary, and ophthalmic nerves are located along the lateral areas of the cavernous sinuses. In the medial areas, there are the internal carotid artery, abducens nerve, and sympathetic plexus.^30^

Blood from the inferior and superior ophthalmic veins, the sphenoparietal sinus, and the middle cerebral veins reaches the cavernous sinus. It drains into the emissary veins that lead to the superior and inferior petrosal sinuses and the pterygoid plexus. Since the anatomically rich venous connections are valveless, infection and thrombus can move forward or backward, making the cavernous sinus prone to sepsis.^30-32^ Although it is prone to sepsis, it should be kept in mind that cavernous sinus thrombosis (CST) may be of aseptic origin. Cases that are septic are called septic CSTs. The mechanism of septic CST is directly related to the embolization of bacteria that triggers thrombosis and infection in the cavernous sinus. The majority of cases are caused by sinusitis.^33^ OIs are not common causes of septic CST and account for less than 10% of all septic CST cases.^34^ Septic CST is extremely rare due to serious OIs. However, recognizing the complication and treating it immediately is extremely important for medical ethics and to prevent any serious complications that may occur.

When septic CSTs were examined microbiologically, it was seen that the majority of them were of bacterial origin. The most common pathogen detected is *Staphylococcus aureus*, followed by Streptococcus species, oral anaerobic flora, and gram-negative bacteria.^35^

In the presence of conditions affecting the cavernous sinuses, anatomical contexts provide important clinical clues. Patients typically present with tachycardia, hypotension, unilateral headache, fever, eye pain, chemosis, periorbital edema, proptosis, ophthalmoplegia, and vision loss.^2^ Ocular symptoms of CST may mimic orbital cellulitis, superior ophthalmic vein thrombosis, carotid-cavernous fistula, orbital apex syndrome, and superior orbital fissure syndrome. Therefore, clinicians should suspect SCST in patients with multiple ophthalmoplegia, proptosis, ptosis, fever, and sensory impairment in the ophthalmic and maxillary regions.^36^ Indispensable for a definitive diagnosis is brain CT, or magnetic resonance imaging.^36^

In the pre-antibiotic era, septic CST had mortality rates as high as 80%-100%. Following the discovery of antibiotics and advances in diagnostic imaging, the incidence and current morbidity/mortality rates of septic CST are estimated to be 20%-30%.^35^ There is a possibility of neurological sequelae in half of the patients, which emphasizes the need for rapid diagnosis and treatment of the disease.^35^

In the treatment of septic cavernous sinus, antimicrobial and antithrombotic treatments should not be ignored, with or without surgical drainage of the relevant area. Depending on the clinical condition of the patient, vancomycin and nafcillin plus a third-generation cephalosporin plus metronidazole and broad-spectrum antibiotics are preferred until the causative microorganism is found in the treatment, which will last intravenously (6-7 weeks) or with somites (3-4 weeks). In developing countries, amphotericin B antifungal treatment is required.^37^ Additionally, steroid use is not recommended in the acute phase of this condition.^38^

Although the guidelines regarding the treatment and process of the disease are not clear, stabilization, acute resuscitation, and treatment of the underlying infection (removal of the tooth under antibiotic therapy if the source is odontogenic) are the main steps.^35^

### Cerebral Abscess

Cerebral abscess (CA) is a focal infection of the cerebral parenchyma.^39^ Severe OIs can spread intracranially in 4 theoretical ways: (1) systemic bacteremia, (2) extension to the cavernous sinus via the facial and pterygoid venous systems, (3) inoculation through invasion of foreign bodies, and (4) lymphatic drainage. It is thought that infections spreading to the cranial area follow the systemic bacteremia route.^2^

An odontogenic origin is unlikely to cause a CA. Although the incidence of CA is extremely low in adult patient groups, it is even less common in the pediatric group.^33^ The incidence of CA is 1/100 000 people per year, and 2%-5% of cases are thought to be due to an odontogenic origin.^40^ Human immunodeficiency virus infections, in which immunosuppression is observed, and organ transplantation, in which the immune system is artificially suppressed, increase the likelihood of CA.^41^

If CA, which is extremely rare, develops secondary to OIs, it most commonly affects the frontal lobe^42,43^; *Streptococcus*, *Fusobacterium*, and *Porphyromonas* are the most commonly isolated bacteria. The most common pathogenic species is the *S. milleri* group.^40^

There is usually a latent period before the clinical manifestation of intracranial involvement, and it begins in approximately 7-11 days.^40,44^ Clinical symptoms vary depending on the number, region, and causative organism of the brain abscess.^43^ If the CA is caused by OI, the first clinical symptoms are fever and headache. Neurological findings are often accompanied, and these changes may vary, the most common being confusion. Seizure, hemiplegia, and cranial nerve palsy are other neurological findings.^43^ Clinically, the complaints are initially mild; It usually starts as a headache and fatigue. In cases where neurological changes are not accompanied, headaches and fatigue will delay the diagnosis, as they are very common in diseases such as colds.^40^ It should be noted that the trio of fatigue, headache, and neurological changes is extremely rare, especially in frontal lobe abscesses, which are the lobes most commonly affected by odontogenic CA. It starts to cause symptoms only when it reaches large sizes.^39^

Computed tomography is a rapid technique for the diagnosis of brain abscesses and clarifies the characteristics of the suspicious lesion. It provides a 3-dimensional image that helps detect the size, number, and location of abscesses. However, examination of soft tissues must be supported by magnetic resonance imaging (MRI), and other pathologies, such as neoplastic lesions, must be ignored.^40^ Magnetic resonance imaging has been recommended as the gold standard for diagnosis and follow-up of treatment.^45^

Treatment of brain abscess consists of drainage (via aspiration) and administration of a broad-spectrum systemic antibiotic.^40^ However, current conservative approaches state that in the presence of a small CA (<2.5 cm), if surgery is contraindicated in patients with no accompanying neurological changes and if the source is clearly identified, medical treatment alone will be sufficient.^46^ Antibiotics will be used both in medical treatment alone and in addition to surgery, and ampicillin, vancomycin, and third-generation cephalosporins (ceftriaxone) are administered intravenously.^40,46^ Since the bacterial load is very high in these cases, high doses must generally be administered. The duration of antibiotic treatment depends on the development of the abscess; it will depend on clinical, biochemical, and radiological improvement. In cases with a normal course, antibiotics are administered for 4-6 weeks.^40^

Innovations in diagnosis and treatment enable this frightening infection to be cured without any sequelae. Although rare, neurological sequelae such as vertigo attacks and loss of memory and/or consciousness may be observed^40^ in studies; the presence of ocular complications, gait disorders, and speech difficulties has been confirmed.^23^ While the mortality rate of brain abscess caused by OI was around 30% years ago, it has decreased to around 14% today due to reasons such as increased access to dental treatment and advances in diagnosis and treatment.^40^

### Sepsis

Sepsis is a potentially fatal organ dysfunction that arises from an aberrant response of the host to an infection. For clinical operationalization, organ dysfunction can be represented by an increase in the sequential [Sepsis-related] Organ Failure Assessment (SOFA) score of 2 points or more, which is associated with an in-hospital mortality greater than 10%. Septic shock should be defined as a subset of sepsis in which particularly profound circulatory, cellular, and metabolic abnormalities are associated with a greater risk of mortality than with sepsis alone.^47^ Rarely is a severe odontogenic infection that advances to sepsis observed. Nonetheless, bacteremia might be more prevalent.^2^

Sepsis can be defined in the presence of confirmed or suspected infectious agents accompanied by 2 or more of the systemic inflammatory response syndrome criteria.^48^ The mentioned criteria are (1) body temperature of <36.0°C and/or >38.0°C; (2) >90 heart beats per minute; (3) >20 breaths per minute or PaCO_2_ <32 mm Hg; (4) White blood cell count >12 000/mm^3^, <4000/mm^3^, or >10% immature (band) forms.

In a study conducted by Wong,^49^ it was reported that 18 of 2790 patients with maxillofacial infection died due to infection. The study found that the most common cause of death was sepsis, with a rate of 55%. Risk factors that may accelerate septic progression in patients with severe odontogenic infection include underweight, immunosuppression, children under 1 year of age, adults over 75 years of age, and drug users.^50^ It is of great importance to diagnose sepsis as early as possible. Sepsis must be identified at the earliest opportunity.

It is recommended to start antibiotic treatment as soon as possible in sepsis and within 1 hour after diagnosis in patients with septic shock. For ideal drug selection, culture collection should be done without delay. Although starting antibiotics quickly increases the chance of survival, the most important thing is to use antibiotics that are effective against the pathogen causing the infection.^51^ It has been reported in the literature that more studies are needed and that combined empiric antimicrobial treatments give better results than single-agent treatment.^52,53^

The necessity and sufficiency of the use of antibiotics are not only related to the spectrum in which they are effective; it is also related to the application technique, adequate dose, and duration. Antibiotics are often given intravenously for 7-10 days, but longer periods of antibiotic use may be indicated in patients with a slow clinical response, in patients who have additional opportunistic fungal or viral infections, or who are immunosuppressed.^54^

In patients with sepsis, organ dysfunctions that are thresholds should also be taken into consideration in drug selection and dose adjustment for antimicrobial treatment.^55,56^ In specific cases, until the exact dose is determined, adjusting the drug and its doses to each patient, adjusting the dose for each patient, and monitoring the doses daily may be helpful for treatment.^57,58^

In the “International Guidelines for the Management of Sepsis and Septic Shock” published in 2021, it is recommended that patients with hypoperfusion or septic shock caused by sepsis be given at least 30 mL/kg IV crystalloid fluid within the first 3 hours after resuscitation.^59^

Patients who demonstrate indications of sepsis should be referred to the local emergency department without delay, so that additional investigations can be conducted and appropriate action can be taken. Prompt intervention may involve the administration of high-flow oxygen, intravenous hydration, and antibiotics.^60^

Prompt transfer of patients presenting with orofacial infections with suspected sepsis to the acute hospital setting for early treatment will improve survival rates. In addition, the patient who seeks the advice of dental professionals may have acquired sepsis for reasons other than odontogenic origins. After receiving a diagnosis of red-flag sepsis, the dentist ought to initiate the basic life support procedure while calling for emergency medical assistance until the arrival of the emergency team. Until the team arrives, the patient should be monitored by administering oxygen at a rate of 15 liters per minute. Early diagnosis and antibiotherapy are crucial for the management of the sepsis.^50^

### Necrotizing Fasciitis

Necrotizing fasciitis (NF) is defined as an aggressive bacterial infection that affects the superficial fascial layer and subcutaneous cutaneous tissues, causing fulminant, destructive, and rapidly progressive necrosis of the affected tissues.^61,62^ Cervicofacial NF, which often results from OI foci, is rare and accounts for only 1%-10% of NF cases.^63-66^ Diabetes, accompanied by a high mortality rate, is an important risk factor in this progression. It has been reported that the death rate in patients with diabetes is almost 3 times higher than the total population.^67^

Clinical and radiographic findings such as OIs are used in the diagnosis of NF, which in its early stages can be confused with a common OI and misdiagnosed as abscess, erysipelas, or cellulitis.^65,68^ In the advanced stages of the disease, small purple dots, dark hemorrhagic blisters, crepitation, complete anesthesia, and dark necrosis of the affected skin become evident, indicating the pathognomonic picture. At this stage, accompanying hypotension, impaired consciousness, and tachypnea indicate a life-threatening situation.^69^ Many researchers have agreed on the importance of early and rapid diagnosis and urgent intervention, as a delay in treatment will result in a significant decrease in the probability of survival.^61,65,70,71^

To improve early diagnosis, researchers have identified 3 stages of NF based on cutaneous features, progressing beyond the visible area of skin involvement. They stated that tenderness to palpation, erythema, swelling, and temperature were the first stages of symptoms. The second stage involves the formation of blisters or bullae, while the last stage is characterized by crepitation and skin anesthesia accompanied by advanced dark discoloration and skin necrosis.^72^ Insufficient definition of the affected tissue edges and sensitivity beyond the limits of the visible area of involvement are clinical features that distinguish early NF from erysipelas or cellulitis.^1,2^

Early surgery, debridement, and antibiotic treatment have been shown to reduce the mortality rate.^73^ In a systematic review showing the importance of treatment timing in necrotizing soft tissue infections, it was stated that the mortality rate was 19% in patients who received surgical treatment within the first 6 hours of admission and 32% in patients who received surgical treatment after 6 hours.^74^ The most important factor in NF is the time from the patient’s application to the surgery. Although CT provides advantages in detecting NF, the time required to obtain the image and the conditions of some hospitals are seen as disadvantages. Facial air or gas, soft tissue edema, or facial contrast images are radiographic findings.^2^

Necrotizing fasciitis may be polymicrobial or due to a single organism. It has been reported that 30 different microorganisms were isolated from cultures taken from patients with odontogenic NF. While classifying the isolated microorganisms, results ranging from *Staphylococcus* and *Streptococcus* species to mixed anaerobic species and, less frequently, bacteria such as *Prevotella* and *Fusobacterium* were encountered.^67^ The main reason for the occurrence of NF, rather than a pathological group, is the coexistence of certain virulence factors that affect the metabolism of polysaccharides and oxygen and have the ability to produce certain destructive enzymes.^71,75^ It should be noted that these factors can only detect a part of the responsible bacterial community in microbiological tests performed in the treatment of odontogenic NF. In cases that do not respond adequately to therapeutic measures, accurate bacterial detection is important for treatment prognosis. Therefore, in the future, culture-based routine microbiological diagnoses need to be supported by culture-independent molecular detection methods.^69^ Molecular detection is important for a rapid diagnosis. However, culture and antimicrobial sensitivity tests are very valuable for appropriate treatment.

The 2 important steps in the treatment of odontogenic NF are surgical exposure of the affected fascia by radical removal of necrotic tissue and subsequent administration of empirical broad-spectrum antibiotic therapy with dose-determined antibiotic therapy according to an antibiogram.^65,76^ While metronidazole, clindamycin, penicillin, and ceftriaxone are frequently used as broad-spectrum antibiotics, dose adjustment may be required according to culture results.^67^

Wounds should be treated with negative wound pressure therapy to avoid being affected by bacterial enzymes and formation of metabolites that cause necrosis. Hyperbaric oxygen therapy may be a useful adjunct in refractory cases. Postoperatively, patients should receive intensive medical care, and at least 1 second-look surgical intervention should be performed the next day to safely eliminate further developing necrosis. In common cases, various interventions are required to prevent the development of new necrosis.^69^

According to intensive care treatment, tracheostomy may be preferred in patients when there is no necrosis in that area. If the area is infected, this procedure should be avoided considering the risk of infection spreading to the mediastinum.^66^ If there is no further necrosis after consecutive surgical interventions, treatment can continue with the burn treatment procedure. While mesh skin grafts can be considered for treatment on large debridement surfaces, healing in small areas limited to the neck can be left to free granulation.^69^ Additionally, airway management and management of hypotension, hypovolemia, and malnutrition may be necessary in patients with odontogenic NF.^67^

### Lemierre’s Syndrome

Thrombophlebitis of the internal jugular vein (IJV), accompanied by a cervical cavity infection that usually extends to the thorax, is defined as Lemierre’s syndrome (LS). It is a complication that can rarely be seen with the progression of complications such as sepsis and septic embolization of the lung secondary to anaerobic infection in the head and neck area, but its clinical course is fatal.^77,78^

While LS is seen in 85% of tonsil and pharyngitis infections, this rate is 2%-3% in OIs and mastoiditis.^79-81^ In a study examining the origin of LS, it was reported that only 8% of the cases examined were of odontogenic origin.^82^ This syndrome is known to be more common in young adults; it is seen at lower rates, especially with the widespread use of antibiotics to treat streptococcal pharyngitis.^78^

While fever, shortness of breath, and submandibular and sublingual edema are reported to be more common in LS cases originating from OI, associated cervical edema, localized pain in the chest, dysphagia, and limitation in mouth opening are also listed among the symptoms.^81,82^ The most common microorganism in its etiology is *Fusobacterium necrophorum*, which causes platelet aggregation due to virulence factors.^80,83^ There are studies in the literature reporting that no specific microorganism can be identified in LS.^84^ However, it is thought that the use of antibiotics in recurrent OIs, false negative results from culture studies, and difficulties in detecting *F. necrophorum* in culture may also cause these results. Additionally, apart from *Fusobacterium*, *Staphylococcus*, *Streptococcus*, *Peptostreptococcus*, and *Bacteroides* are among the causative microorganisms.^80,83,84^

Although pharyngitis and tonsillitis are frequently seen in patients who develop LS, reports of patients without upper respiratory tract infections have also been reported.^78,79,85^ Criteria for diagnosing LS are listed as the primary infection area localized in the head and neck region, thrombosis/thrombophlebitis or metastatic lesions observed in the IJV or the veins in the region, and the isolation of *F. necrophorum* from a sterile area or its detection in blood culture.^86^

Treatment of LS takes longer because the course of the disease is endovascular. It seems that the use of metronidozole is common in antibiotic treatment, which is preferred to affect anaerobic bacteria, especially *F. necrophorum*. It is reported that the treatment takes an average of 42 days (between 7 and 48 days).^87^ Although there is no definitive acceptance of its use as an adjunct to treatment, anticoagulant use is also possible for different periods of time. The reason for this is thought to be the positive effect of anticoagulants in the treatment of IJV thrombosis.^88^ A case history report in the literature reported that the patient was treated in the hospital for 2 months, 20 days of which were in intensive care, and that the patient was also given anticoagulants along with broad-spectrum antibiotics.^78^

Despite the low incidence of OI-induced LS, quick diagnosis and management of this syndrome is critical owing to its progressive nature. In cases originating from OIs, the mechanism of development of this condition is the spread of OI to the pharyngeal cavities. Treatment includes control of the origin of infection and antibiotic treatment.^2^

## Conclusion

Odontogenic infections typically affect only the adjacent tissues or alveolar bone, which are in close proximity to the teeth. Nevertheless, infection may invade deep fascial spaces. Severe complications may arise in the case of infections that involve these fascial spaces and exhibit more extensive dissemination. Diagnosis and treatment of OIs in a timely manner prevents complications. Dental professionals play a critical role in the prevention and assessment of OIs, particularly those that are progressive and difficult to treat and identify.
